# Assessment of the Tissue Response to Modification of the Surface of Dental Implants with Carboxyethylphosphonic Acid and Basic Fibroblastic Growth Factor Immobilization (Fgf-2): An Experimental Study on Minipigs

**DOI:** 10.3390/biology10050358

**Published:** 2021-04-23

**Authors:** Javier Aragoneses, Ana Suárez, Nansi López-Valverde, Francisco Martínez-Martínez, Juan Manuel Aragoneses

**Affiliations:** 1 Department of Medicine and Medical Specialties, Faculty of Health Sciences, University of Alcalá, 28801 Madrid, Spain; javias511@gmail.com; 2 Department of Preclinical Dentistry, School of Biomedical Sciences, Universidad Europea de Madrid, Villaviciosa de Odón, 28670 Madrid, Spain; 3 Department of Surgery, Instituto de Investigación Biomédica de Salamanca (IBSAL), University of Salamanca, 37007 Salamanca, Spain; nlovalher@usal.es; 4 Orthopaedic and Trauma Service, Virgen de la Arrixaca University Hospital, El Palmar, 30120 Murcia, Spain; fmtnez@gmail.com; 5 Dean of The Faculty of Dentistry, Universidad Alfonso X El Sabio, 28691 Madrid, Spain; jmaragoneses@gmail.com

**Keywords:** carboxyethylphosphonic acid, fibroblast growth factor, dental implants, minipigs

## Abstract

**Simple Summary:**

This study aimed to evaluate the efficacy of treating the surface of dental implants with carboxyethylphosphonic acid for the immobilization of FGF-2, the influence of FGF-2 on cortical bone in close contact with dental implants, new bone formation around dental implants in the presence of FGF-2 and the influence of FGF-2 on the interthread bone area of dental implants during the healing period after insertion.

**Abstract:**

The aim of this study was to evaluate the effect of implant surface treatment with carboxyethylphosphonic acid and fibroblast growth factor 2 on the bone–implant interface during the osseointegration period in vivo using an animal model. The present research was carried out in six minipigs, in whose left tibia implants were inserted as follows: eight implants with a standard surface treatment, for the control group, and eight implants with a surface treatment of carboxyethylphosphonic acid and immobilization of FGF-2, for the test group. At 4 weeks after the insertion of the implants, the animals were sacrificed for the histomorphometric analysis of the samples. The means of the results for the implant–bone contact variable (BIC) were 46.39 ± 17.49% for the test group and 34.00 ± 9.92% for the control group; the difference was not statistically significant. For the corrected implant–bone contact variable (BICc), the mean value of the test group was 60.48 ± 18.11%, and that for the control group, 43.08 ± 10.77%; the difference was statistically significant (*p*-value = 0.035). The new bone formation (BV/TV) showed average results of 27.28 ± 3.88% for the test group and 26.63 ± 7.90% for the control group, meaning that the differences were not statistically significant (*p*-value = 0.839). Regarding the bone density at the interthread level (BAI/TA), the mean value of the test group was 32.27 ± 6.70%, and that of the control group was 32.91 ± 7.76%, with a *p*-value of 0.863, while for the peri-implant density (BAP/TA), the mean value of the test group was 44.96 ± 7.55%, and that for the control group was 44.80 ± 8.68%, without a significant difference between the groups. The current research only found a significant difference for the bone–implant contact at the cortical level; therefore, it could be considered that FGF-2 acts on the mineralization of bone tissue. The application of carboxyethylphosphonic acid on the surface of implants can be considered a promising alternative as a biomimetic coating for the immobilization of FGF-2. Despite no differences in the new bone formation around the implants or in the interthread or peri-implant bone density being detected, the biofunctionalization of the implant surface with FGF-2 accelerates the mineralization of the bone–implant interface at the cortical level, thereby reducing the osseointegration period.

## 1. Introduction

The macroscopic and microscopic designs of dental implants are of great relevance. The microscopic design (implant surface) is considered more important in the initial phases of osseointegration and in initial loading, while the macroscopic design (implant design) is more important in the mature phases of loading [1,2]. The design of a dental implant is one of its main characteristics, since critical factors such as load distribution depend on it and are intimately related to implant survival and the maintenance of long-term osseointegration [3]. Other design parameters that affect the load distribution have also been observed, such as the diameter (width, or smallest dimension of the implant) and the length (length, or longest dimension of the implant) of the bone–implant interface, as well as the depth, the shape and the thread pitch of the turn in threaded implants [4]. The surface of a biomaterial is the only part that remains in contact with the biological environment; therefore, it plays a crucial role in the biological response of bone tissue. Characteristics such as the composition of the surface and its topography and roughness as well as its surface energy affect the mechanical stability of the bone–implant interface and osseointegration at the histological level [5,6]. The quality of the implant surface increases roughness or develops microcavities that can favor the union of macromolecules on the implant surface and bone and will determine the reaction of the bone tissue to the implantation in the oral cavity [7]. The application of treatments on the surface of implants in order to increase the roughness of said surface has been widely studied and has shown better osseointegration in the short and medium term [8,9,10]. In addition, despite a success rate of 95% in the short term, late implant failures are associated with peri-implantitis [11,12,13]. Surface bioactivation is a biochemical method of surface modification, whose objective is based on the immobilization of proteins, enzymes or peptides that induce a specific cellular response at the bone–implant interface. To modify this type of surface, organic components are used, which are known to create a response in the bone and promote cell adhesion, such as identification of the Arg-Gly-Asp (RGD) sequence, a mediator of cell binding with plasma proteins and extracellular matrix proteins (fibronectin, vitronectin, type I collagen, osteopontin or bone sialoproteins) [14,15,16,17]. The development of biocompatible layers that attempt to mimic the adhesion of osteoblasts to obtain better and faster osseointegration is an ongoing investigation. In recent years, different studies have been carried out aimed at treating the surface of TiCP implants to achieve a rougher surface that allows the possibility of anchoring bioactive substances that will improve the tissue integration of implants. One of these techniques consists in treating the implant surface with carboxyethylphosphonic acid. Carboxyethylphosphonic acid, also known as 3-phosphonopropionic acid (HO_2_C-CR_1_H-CR_2_H-PO_3_H_2_), is characterized as a powerful corrosion inhibitor. Phosphonic acid molecules can form stable bonds with passivated metal oxides, such as aluminum oxide (Al_2_O_3_) or titanium oxide (TiO_2_), producing an organic monolayer on which modifications could be made to improve cell adhesion and the biocompatibility of the surfaces of dental implants [18]. Coating the surfaces of implants with bioactive molecules to change their properties can positively modulate the biological response; covalently linked hyaluronan dental implantation following interfacial interactions resulted in a satisfactory split-mouth clinical outcome at 36 months in a follow-up study [19].

Following this line of research, this work proposes the binding of fibroblast growth factors (FGF-2) on the surface of dental implants to improve bone–implant union and the possibility of increasing its speed of osseointegration based on the ability of FGFs to increase angiogenesis in vivo, therefore playing a crucial role in wound healing [20]. They have been shown to control the switch between adipocytes and the differentiation of osteoblasts in the mesenchymal cells of the bone marrow stroma [21]. As has been shown in recent studies, FGFs are proteins with osteoinductive properties, which are actively involved in osteoblastogenesis [20,22]. The present study is an experimental research work on an ‘in vivo’ animal model, the purpose of which is to assess the bone tissue response in the presence of two groups of implants (one group with standard surface treatment and the other with a carboxyethylphosphonic acid surface treatment and immobilization of FGF-2). For this purpose, the interthread and peri-implant bone density as well as bone–implant contact and neoformation were evaluated histomorphometrically, which are parameters studied to evaluate the behavior of new coatings.

## 2. Materials and Methods

### 2.1. Animal Models

This research was carried out on 6 minipigs of the Landrace breed (Subspecies: Sus Scrofa Domestica), aged between 6 and 8 months and weighing between 20 and 25 kg, from an experimental animal production farm (Distrizoo Animals SL, Madrid, Spain). This experimental research in pigs was presented for Internal Regulations of the Ethics and Animal Welfare Committee on 4 October 2012 and approved on 31 January 2013 by the Ethics Committee in Animal Experimentation (CEEA) of the University Hospital Puerta de Hierro Majadahonda (Madrid, Spain) and the Puerta de Hierro Majadahonda Research Institute (IDIPHIM). Additionally, it followed the current international regulations on experimental animals: Royal Decree 1201/2005 of October 10 (86/609/CEE and ETS 123) on the protection of animals used in experimentation and for other scientific purposes, as well as Council Directive 86/609/EEC of 24 November 1986 on the approximation of the laws, regulations and administrative provisions of the Member States regarding the protection of animals used in experimentation and other scientific purposes.

### 2.2. Implants and Surface Treatment

For this research, the surgical procedure was performed by the same oral surgeon, and two study groups were created. A total of 16 dental implants of grade IV titanium, 4 mm in diameter and 10 mm in length, with an internal conical connection of 11° (Surgimplant IPX Galimplant^®^, Galimplant S.L.U., Sarria, Lugo, Galicia, Spain), were inserted. All the surgeries were performed on the same day. The implants were divided into two groups: the control group (SE), with a standard Company SLA surface treatment, and the test group (SP), with a surface treatment of carboxyethylphosphonic acid and immobilization of FGF-2.

For the surface treatment, the implants were subjected to an immersion process in a mixture with 50 mL tetrahydrofuran (Uvasol^®^, Madrid, Spain) and 55 mg carboxyethylphosphonic acid. The immersion was maintained for 24 h at a temperature of 76 °C. After finishing the immersion process, the implants were rinsed with deionized water, thermal dryed, and submerged in a dilution consisting of 5 mL distilled water, 175 mL of ethyl-3-(3-dimethylaminopropyl) carboxyamide, and 54 mg of N-hydroxysulfamide for 15 min at room temperature. Afterward, the implants were gently washed with deionized water and included, individually, into 0.5 mL microcentrifuge tubes (Eppendorf Tubes^®^, Eppendorf AG, Hamburg, Germany) in a prepared stock solution consisting of distilled water and FGF-2 at a concentration of 8 µg/mL. The stability of the pH was checked (pH 7) using a pH meter (MP230, Mettler Toledo^®^, Barcelona, Spain) and the implants were incubated in the solution in an incubator at 37 °C for 1 h (Galaxy^®^ 170, Eppendorf AG, Hamburg, Germany).

After the chemical manipulation of the implants, they were introduced into ultrasonic tanks to eliminate the impurities present on the implant surface packed in laminar flow cabinets under a sterile atmosphere (without any type of microbial life or contaminant) and sterilized by gamma radiation at a dose of 25 KGy. The implants were sealed under the manufacturer’s guarantee of sterility (Galimplant^®^, Sarria, Lugo, Galicia, Spain). The entire handling process was carried out in a sterile environment and field.

Before the surgical intervention, each implant was stored in a separate opaque envelope. Then, each of the implants was introduced according to its type of surface in two large sachets to avoid mixing so that the 8 test implants would remain in one sachet and the 8 control implants in another sachet. For the coding of the samples, letters were assigned to each envelope. The coordinator placed a label with the identifying letter (hidden) on each envelope according to the surface treatment that it presented. Therefore, the 16 implants remained in two envelopes: an envelope with 8 control surface implants with the identification letters SE, and an envelope with 8 test surface implants with the letters SP.

Each of the experimental animals used in this study had a dossier (C1, C2, C3, C4, C5 and C6). Inside each of the dossiers, the implants were included; of the 6 dossiers, 4 housed an envelope with 3 implants from the same group, while in the other 2 dossiers were envelopes with 2 implants from the same group of study chosen at random. Each of the envelopes included in each dossier corresponded to the implants to be placed in the left posterior tibia (TI) of each experimental animal.

**Figure 1:**
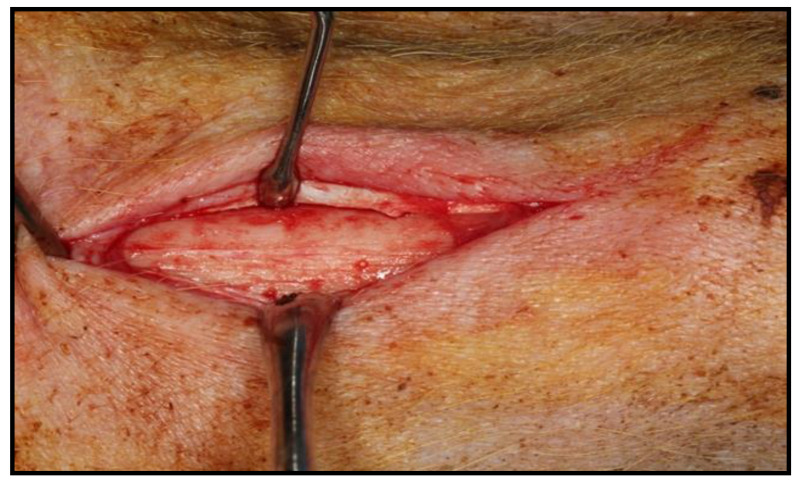
Mucoperiosteal detachment exposing the bone surface.

Both the surface of the implants to be incorporated into each dossier, and the implants and the positions in which they should be placed (Mesial ‘M’, Center ‘C’ and Distal ‘D’) in the left tibia of each animal (Figure 1) were randomly assigned.

### 2.3. Surgical Intervention

In the 18 h before the intervention, the animals remained on a solid food fast, allowed to consume water up to 6 h before starting the surgery, to guarantee the smallest possible volume of gastric content and, thus, avoid possible complications during the procedure, such as the regurgitation or aspiration of gastric contents.

Premedication was performed intramuscularly in the lateral part of the neck (at the level of the trapezius and cleido-occipital muscles), using a 20 G needle (BD Microlance^®^, Becton Dickinson, Franklin Lakes, NJ, USA) and a 5 mL syringe (BD Plastipak^®^, Becton Dickinson, Franklin Lakes, NJ, USA). Concentrations used were as follows: ketamine (Ketonal 50^®^, Richmond Vet Pharma, Buenos Aires, Argentina) at a dose of 5 mg/kg and midazolan (Dormicum^®^, Roche S.A., Basilea, Switzerland), at a dose of 0.5 mg/kg. Medetomidine (Medetor^®^, Virbac, Carros, France) was used at a dose of 0.2 mg/kg. Finally, atropine (Atropina^®^, Pharmavet, Bogotá, Colombia) was used at a dose of 0.3 mg/kg.

**Figure 2:**
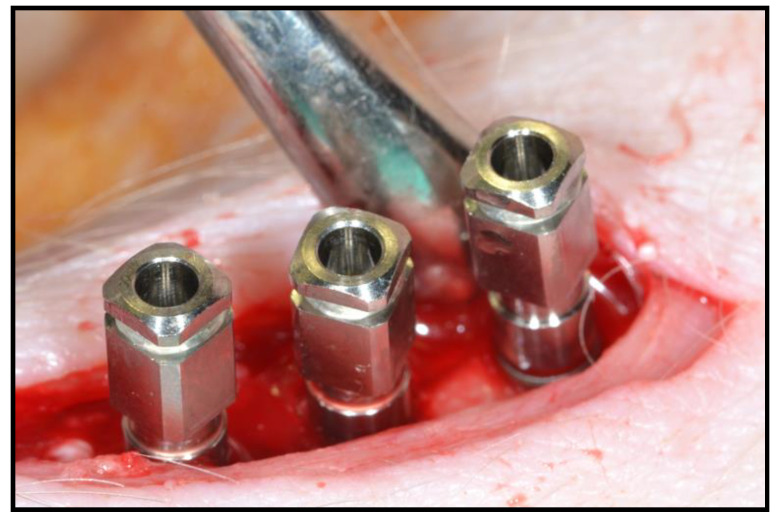
Placement of the 3 implants in the tibia with a separation of 5 mm.

Once sedated, the hind legs of the animal were immobilized to shave the area to be treated (internal face of the left tibia of the hind limbs). When all the animals were prepared for the intervention, a veterinarian (who had no knowledge about the trial) randomly chose the order of surgery for the 6 pigs. To later differentiate them, a tattoo of Roman numerals (I, II, III, IV, V, and VI) was made using Chinese ink on the right ear of each animal, with the number I corresponding to pig 1 (Dossier 1), etc. For the induction of general anesthesia and for endotracheal intubation, propofol (Diprivan^®^, AstraZeneca, Cambridge, UK) was administered intravenously. The used doses of propofol were 2–6 mg/kg for induction and 0.2–0.4 mg/kg/min for maintenance. The animals were connected to an automatic ventilator (Oxylog^®^ 3000, Dräguer, Lübeck, Germany) as well as to a capnograph (Oxylog^®^ 3000, Dräguer, Lübeck, Germany). In addition, throughout the surgical procedure, each animal was monitored with an electrocardiogram evaluation (GradyVet ECG 1000, Grady Medical Systems, Murrieta, CA, USA) and temperature control. Epidural anesthesia was carried out with bupivacaine 0.75% (Bupinex^®^, Richmond Vet Pharma, Buenos Aires, Argentina) at a dose of 4.5 mL/Kg and fentanyl (Fentanilo^®^, Kilab, Buenos Aires, Argentina) at a dose of 0.005 mg/Kg. In addition, loco-regional anesthesia in the dermis of the area to be intervened was used; an infiltrative technique was applied with 4% articaine and adrenaline in a ratio of 1:100,000 (Ultracain^®^, Normon, Madrid, Spain). In each of the posterior left tibiae of the pigs, 2 or 3 implants were placed. The surgical drilling protocol that followed was the conventional osseointegrated implant placement recommended by the manufacturer. An implant motor (Implantmed SI-923, W&H, Burmoos, Austria) was used with a micromotor (AM-25 E RM, W&H) and a 20:1 reduction contra-angle (WS-75 LG, W&H) from the same brand, with external irrigation through a dispenser (Omnia^®^, Fidenza, Italy) and physiological saline (Vitulia^®^ 0.9%, Barcelona, Spain) (Figure 2). After surgery, each animal was administered antibiotic coverage to avoid infection of the surgical wound. The antibiotic used was amoxicillin (Clamoxyl^®^, Pfizer, New York, NY, USA) at a dose of 1.5 g, prepared as a solution for injection intramuscularly for a period of 5 days. The opioid used was buprenorphine (Buprex^®^, Quintiles, Danbury, CT, USA), administered intramuscularly at a rate of 0.01–0.04 mg/kg, every 6–8 h. Four weeks after the implants were placed, all animals were sacrificed.

### 2.4. Sample Praparation

The samples were kept in 10% formalin for at least 15 days before studying them. Subsequently, their processing was continued, following the protocol proposed by Donath and Breuer in 1982 [23]. Plastic infiltration was carried out by mixing glycolmethacrylate (Technovit 7200^®^, Heraus Kulzer, Werheim, Germany) and benzoyl peroxide (BPO^®^, Heraus Kulzer, Werheim, Germany) with ethyl alcohol at different concentrations, with two final infiltrations in pure glycolmethacrylate. These steps were carried out under constant agitation with a reciprocating shaker (SM30, Edmund Bühler, Germany), as in the dehydration process. For histological analysis, specimen preparations were photographed using a 40× digital camera (Stylus SP-820UZ, Olympus, Tokyo, Japan) employing a motorized light microscope (BX51, Olympus, Tokyo, Japan). The photographs were combined using a computer program (Cell Sens Dimensions, Olympus, Tokyo, Japan) to obtain high-resolution images to evaluate the response of the bone tissue.

**Figure 3:**
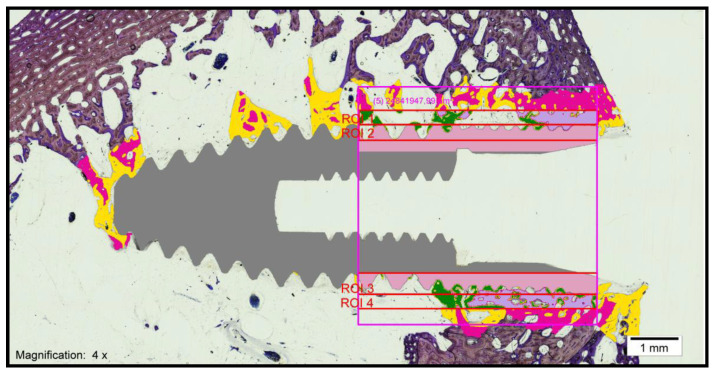
Treatment of the images using the Adobe Photoshop CS3 program (San Jose, CA, USA) and a digitizing tablet (Intuos 4 large, Wacom, Saitama, Japan).

After treating the images for the histomorphometric analysis, the bone area was quantified concerning the total tissue area of each sample using the same software (Cell Sens Dimensions, Olympus, Tokyo, Japan). The measurements made in this study for analysis were based on the study by Kuchler et al. (2013) [24], as follows:

- Bone–implant contact (BIC): percentage of the implant surface in direct contact with the bone (Figure 3).

- Corrected bone–implant contact (BICc): Defined as the length of bone in direct contact with the implant surface concerning the partial perimeter of the implant—that is, eliminating the implant portions not surrounded by bone in the coronal and apical parts of the implant. In this way, implant sections surrounded by tissues other than bone are discarded.
- New bone formation (BV/TV) (bone volume/total volume): Defined as the area of new bone formed after dental implant placement, expressed as a percentage (%). Quantifies the volume of mineralized bone and is generally located between the implant threads and at a distance of up to 300 microns around the implant (peri-implant area).
- Interthread bone density (BAI/TA): Defined as the area of bone within the threads of the implant divided by the total area of tissue within them. The final result is multiplied by 100 and expressed as a percentage. In summary, this is the percentage of bone within the interthread portion.
- Peri-implant bone density (BAP/TA): Defined as the bone surface that grows along the length of the implant within the thread in relation to the total available space and the amount of bone in relation to the total surface to a distance of 0.3 mm from the implant. A rectangle, 5 mm long and 300 microns wide, is used in histomorphometry, formed by a line that joins the peaks of the implant threads (parallel to the peak implant thread). Therefore, the peri-implant bone is defined as the area of bone within the rectangle divided by the total area of tissue within the same.

To obtain this measurement, the digitized images were treated using software (Adobe Photoshop CS3, San Jose, CA, USA) and a digitizing tablet (Intuos 4 large, Wacom, Saitama, Japan), whereby the bone surrounding the implants was marked in yellow in the digitized images. Once the images were processed, the bone area was quantified in relation to the total tissue area of each sample using software (Cell Sens Dimensions, Olympus, Japan)

### 2.5. Statistical Analysis

The statistical analysis of this research was carried out in two different phases:

Descriptive statistics: The usual descriptive statistics were calculated for each variable, which included the arithmetic average, median, typical deviation, variance, range and typical error. The arithmetic mean values were expressed through a confidence interval (95%), in which the lower and upper values of the said interval were recorded. In the descriptive study, comparative graphs of boxes and bars were attached for each of the variables involved in the study to be able to visualize, in an approximate way, what, in the second phase, was calculated as an inferential study.

Inferential statistics: A significance level of 5% (α = 0.05) was used. The data for each implant, including the bone–implant contact (BIC), corrected bone–implant contact (BICc), new bone formation (BV/TV), interthread bone density (BAI/TA) and peri-implant bone density (BAP/TA), were statistically analyzed by subjecting the variables to tests to detect the associated probability distributions.

Two test methods were applied to detect whether the datasets followed normal probability distributions:

A: Kolmogorov–Smirnov and Shapiro–Wilk tests to contrast the hypothesis of the normality of the population scores.

B: Q–Q normality graphs, in which each observed value was paired with its expected value, the latter coming from a normal distribution.

In the assumption of detection of normality in the sample distribution, the means were compared using the t-test for two independent variables or the one-way ANOVA procedure. To interpret the results of the comparisons quantitatively, a *p*-value of 0.05 was established, and the confidence interval for the difference of means was used.

## 3. Results

### 3.1. Histomorphometric Analysis of the Samples

#### 3.1.1. Control Group

**Figure 4:**
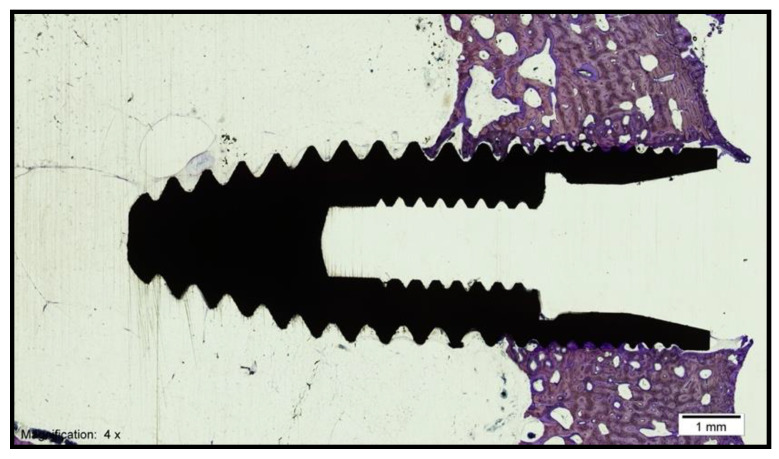
Histological section of an implant from the control group (magnification × 4).

The histological analysis under the light microscope of the longitudinal sections of the samples obtained in the control group (standard surface) revealed the formation of new bone tissue in contact with the implant in the valley and implant thread regions. Additionally, contact between the bone and the implant was observed without signs of interposition of fibrous tissue, with interrupted medullary spaces at the bone–implant interface (Figure 4)

#### 3.1.2. Test Group

**Figure 5:**
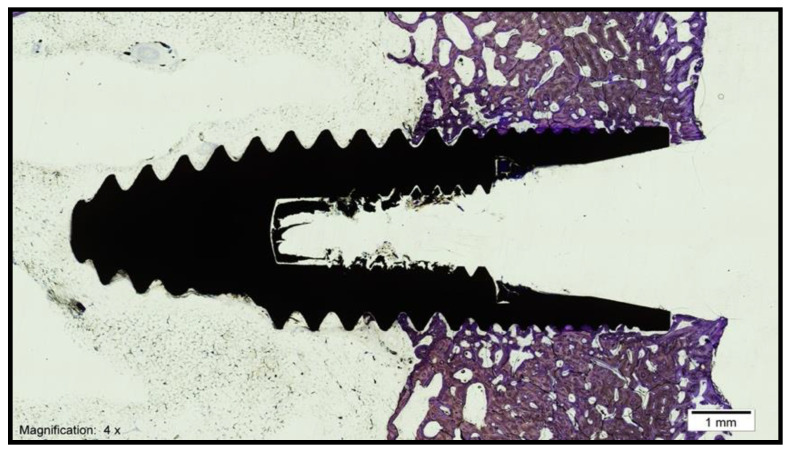
Histological section of an implant from the test group (magnification × 4).

The histological analysis under the light microscope of the longitudinal sections of the samples obtained in the test group revealed the formation of new compact bone tissue. Furthermore, intimate contact between the bone and the implant was observed without signs of fibrous tissue formation, with interrupted medullary spaces at the bone–implant interface (Figure 5). After treating the images for the histomorphometric analysis, the bone area was quantified for the total area of tissue of each sample. For the evaluation of the percentage of bone–implant contact (BIC) or bone integration, new bone formation and bone density, as well as to obtain homogeneous measurements, a 5-mm wide working frame was established on the implant, with one of the sides superimposed on the implant shoulder, excluding the medullary regions in proximity to the cortical bone. A color code was also established for the identification of the different tissues (gray: implant; yellow: new bone; pink: native bone). These measurements were made using the same computer program (Cell Sens Dimensions, Olympus, Tokyo, Japan) and the same digitizing tablet (Intuos 4 large, Wacom, Saitama, Japan) that were previously used for the analysis of the photographs.

### 3.2. Descriptive Statistics

The Kolmogorov–Smirnov and Shapiro–Wilk tests showed that the data fit a normal probability distribution model. Table 1 shows a summary of the BIC variable results in the test and control groups.

**Figure 6:**
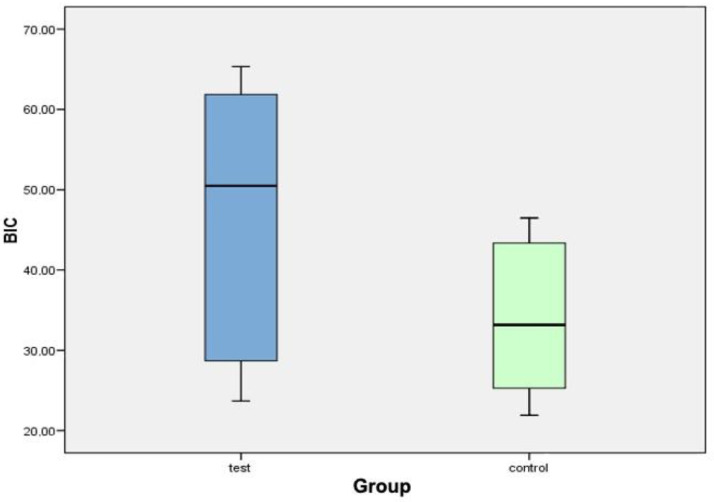
Box diagram corresponding to the BIC variable.

Figure 6 shows a box diagram corresponding to the variable BIC. The central line of each box expresses the value of the median (test value: 50.49%; control value: 33.18%). The mean ± standard deviation in the test group (46.39% ± 17.49%) is higher than that in the control group (34% ± 9.92%). From the data analysis, it can be concluded that no statistically significant differences were found for the BIC variable between the test group and the control group.

Table 2 shows a summary of the BICc variable results in the test and control groups.

**Figure 7:**
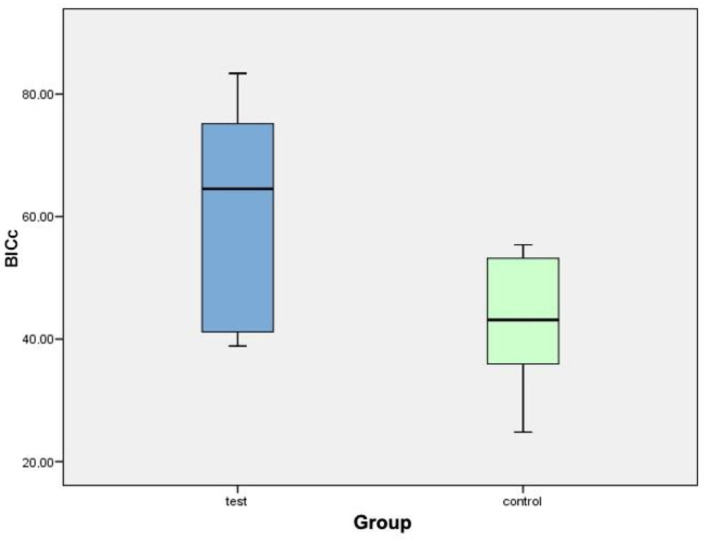
Box diagram corresponding to the BICc variable.

Figure 7 shows a box plot corresponding to the variable BICc. It can be seen how the values of the test group are generally higher than the values of the control group. The central line of each box expresses the value of the median (test value: 64.50%; control value: 43.13%). The average values are higher in the test group. The mean ± standard deviation in the test group (60.48% ± 18.11%) is higher than that in the control group (43.08% ± 10.77%). After analyzing the data, it can be concluded that there were statistically significant differences for the BICc variable between both groups.

Table 3 shows a summary of the of the BV/TV variable in the test and control groups.

**Figure 8:**
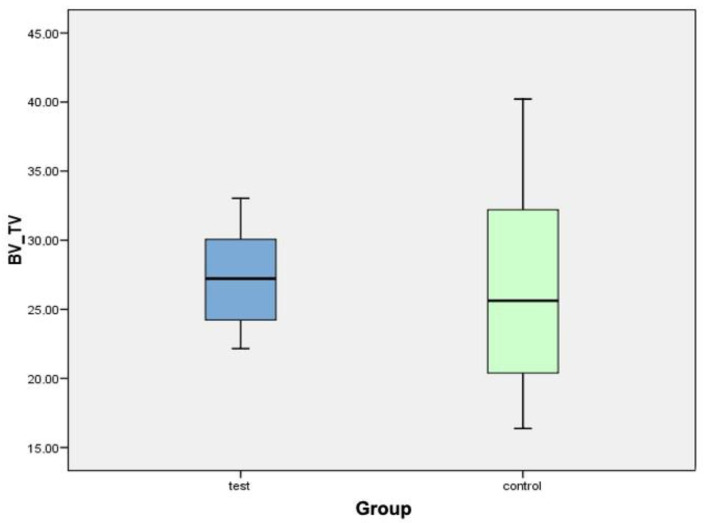
Box plot corresponding to the BV/TV variable.

Figure 8 shows a box plot corresponding to the BV/TV variable. The central line of each box expresses the value of the median (test value: 27.22%; control value: 25.64%). The mean in the test group (27.28% ± 3.88%) is slightly higher than that in the control group (26.63% ± 7.90%). After analyzing the data, it can be concluded that there were no statistically significant differences for the BV/TV variable between both groups.

Table 4 shows a summary of the of the BAI/TA variable in the test and control groups.

**Figure 9:**
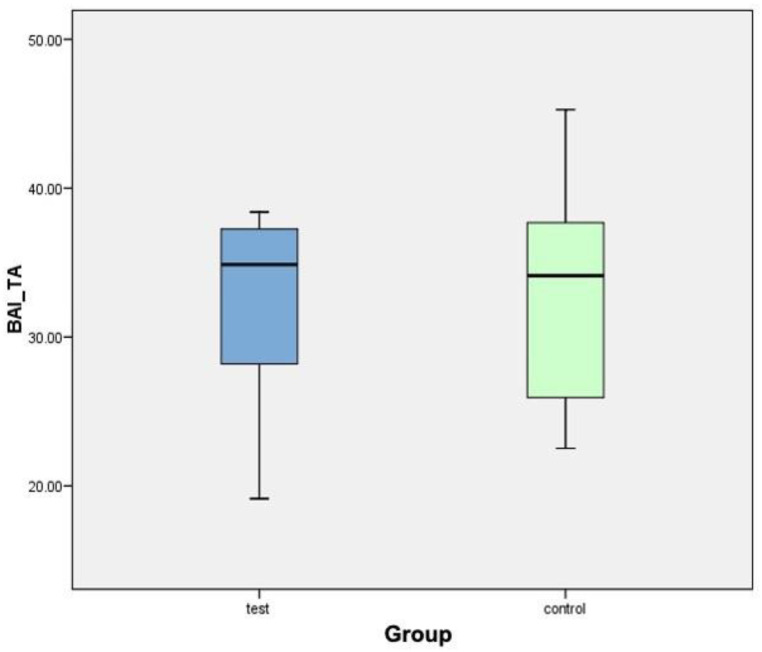
Box plot corresponding to the variable BAI/TA.

Figure 9 shows a box plot corresponding to the variable BAI/TA. The central line of each box expresses the value of the median (test value: 34.88%; control value: 34.12%). The mean in the test group (32.27% ± 6.70%) is slightly higher than that in the control group (32.91% ± 7.76%). After analyzing the data, it can be concluded that there were no statistically significant differences for the variable BAI/TA between the test group (FGF surface) and the control group (standard surface).

## 4. Discussion

The objective of these investigations was to promote the osseointegration mechanism with the formation of bone tissue more quickly and in a greater quantity, the purpose of which is to confer greater stability during the healing process, which also allows for the quicker loading of the implant [25,26]. No studies have been found in the scientific literature evaluating the application of carboxyethylphosphonic acid on the surface of implants with the immobilization of basic fibroblast growth factors in a stimulation of the osseointegration of dental implants in vivo. In the present study, it was possible to immobilize FGF-2 molecules through a covalent bond on the surface of a dental implant previously treated with carboxyethylphosphonic acid. The immobilization of FGF-2 is intended to accelerate the repair process by activating the synthesis of bone-forming cells, a process known as osteoinduction. For this reason, the immobilization of FGF-2 on the surface of dental implants plays an important role in the formation and early development of bone, since its signaling regulates the expression of several genes related to the formation of bone tissue and is involved in the proliferation and differentiation of osteogenic cells [27]. Similar results were obtained by Mamalis et al. [28] in their in vitro study. They chemically modified the rough surface of implants, discovering an upregulation of osteoblastic differentiation and the suppression of osteoclastogenesis regulating the RANKL/RANK/OPG transcriptional axis. Fibroblastic growth factors are pleiotropic growth factors; that is, they intervene in a multitude of biological processes, such as the regulation of cell proliferation, migration, adhesion and differentiation of different tissues, such as epithelial tissue, soft connective tissue, nervous tissue and bone tissue [17,29,30,31]. As previously described, FGF generally stimulates cell proliferation [32], while melatonin is capable of promoting cell differentiation and the mineralization of bone tissue [33,34,35]. To examine the effect of FGF-2 on osseointegration, the authors determined the length of BIC in the measurement regions (zone A, zone B, zone C and zone D). The results obtained in the control group were very low values in zone D at both 4 and 8 weeks. This suggests that the contact seemed to come from the area of the existing bone (zone A) to the side closest to the implant (zone D), where the contact is acquired more slowly. In the test group, the total contact length (all zones) was significantly greater than that in the control group at 4 and 8 weeks. It was observed that the contact with area D (the area closest to the implant) was greater than that in the control group. This may be due to the fact that FGF-2 stimulates the production of osteogenic cells at this level, thus favoring contact osteogenesis. There is a great difference in the result of the BIC variable analyzed by these authors (test: 88.02%; control: 76.37%) with respect to those obtained in the study of this thesis (test: 46.39%; control: 34.00%). It is likely that this large difference is due to the healing time of the implants, since in the present study, the sacrifice of the animals occurred at 4 weeks, while in Carr’s study [36], it was performed at 3 months. In the present investigation, there was only reference to bone healing one month after implant placement; therefore, it could only be assessed at a point in time of bone tissue healing, but the course of healing could not be observed. Therefore, it would be interesting to assess the trend adopted by the two study groups at 2 and 8 weeks. In evaluating the effect of FGF-2 on the surface of the implants in the test group, it could be the case that, at 2 weeks, there was an osteoclastic stimulus that was neutralized at 3–4 weeks with the formation of new bone, equalizing, at 8 weeks, the BIC with the control group, or that just the opposite occurred—that, at 2 weeks, a powerful osteoblastic stimulus was produced with a very intense bone formation that, over time (4 weeks), was neutralized and lost more and more efficiency (8 weeks). In summary, as observed in these studies, bone remodeling occurs earlier in trabecular bone (7 days) than in cortical bone (28 days), which, in turn, leads to faster peri-implant regeneration in trabecular bone, but it should not be forgotten that the cortical tissue, being less porous and having a higher density, provides greater fixation to the implant [36,37]. With the idea of this premise, in the present study, the contact between the implant surface and the cortical bone was evaluated to verify whether the surface treatment used in the present investigation influences the mineralized tissue in the induction of greater integration. For this, only the mineralization of the cortical tissue was considered, and not the trabecular one. Therefore, in observing the statistically significant data obtained for the BICc parameter in the test group compared to the control of the present study, it can be considered that FGF-2 has a positive effect on the stimulation of the mineralization of cortical bone. Okakazi’s study [38] already showed that, at 2 weeks and at 5 weeks, the bone mineral content was double that in a normal bone remodeling process. No studies have been found in the scientific literature that evaluate the effect of fibroblast growth factors for the study of the BICc parameter; therefore, the results of this study cannot be contrasted with other previous investigations. In summary of these observations, it could be considered that bone formation occurs within the first 4 weeks, a period coinciding with the healing period of the implants in the investigation of the present study. It was also observed in the study by Simion et al. [39] that, after 30 days, new bone formation is reduced, and this new bone is replaced by lamellar bone, which becomes much more evident at 90 days. Therefore, for the current research, it is assumed that the advantage of applying FGF-2 on the surface of the implants lies in its effects on osteogenesis, since it presents a proliferative effect on osteoblasts and improves bone production by increasing the number of cells available to synthesize collagen [40]. The idea was to create an implant surface that provides better conditions for the attachment of osteoprogenitor cells to improve bone formation around the implant. Furthermore, in the present study, with the modification of the surface of the implants of the test group through the application of carboxyethylphosphonic acid and the immobilization of FGF-2, we intended to obtain contact osteogenesis. In general, contact osteogenesis forms bone tissue at a 30% faster rate than distant osteogenesis does [41]. In the present investigation, BV/TV is defined as the area of new bone formed after dental implant placement, expressed as a percentage (%). It quantifies the volume of mineralized bone and is generally located between the implant threads and at a distance of up to 300 microns around the implant (peri-implant area). The results obtained by Keiichi et al. [42] at 12 weeks with FGF (28.7 ± 5.5%) are similar to those found in this study at 4 weeks (27.28 ± 3.88%); if the animal model (rats vs. pigs) is also compared, rats show a faster bone metabolism than pigs do. Therefore, with these data, it can be concluded that, in the current study, no statistically significant differences were found between the two groups (test vs. control), and a longer healing period may be necessary. In the present investigation, we tried to histomorphometrically evaluate the bone density and both the interthread (BAI/TA) and the peri-implant (BAP/TA) area of a new surface coating with the application of carboxyethylphosphonic acid and the immobilization of FGF in the dental implants. It has been observed, when reviewing several studies [36,39,40,41,42,43,44], that when evaluating the integration of dental implants with a new surface treatment, the most analyzed variable is BV/TV as a volume of interest of the bone surrounding the bone implant; that is, most authors assess density based on the BV/TV parameter without performing interthread (BAI/TA) and peri-implant (BAP/TA) bone density measurements. Using the same study model to evaluate interthread bone density, Muñoz et al. [44], in 2012, investigated whether the application of melatonin and growth hormone influenced the osseointegration of dental implants. The variations between humans and other animal species are evident, as well as the location in which dental implants are inserted and the state of the host tissue, since they are seldom considered in a rational way. Therefore, it is of great importance to develop and select suitable experimental models at all levels. For this research, minipigs were selected, since before developing a clinical trial in humans, it is necessary to test it in animals, and the model recommended is usually these pigs due to their similarity in bone structure [44]. It is considered that this species can represent human bone tissue with respect to morphology, composition [45] and microstructure [46] as well as its remodeling [47] and mineral density characteristics [48]. However, the trabecular bone tissue of minipigs is denser than that of human bone, since the areolar cavities that make up the network of bone lamellae and where the bone marrow is housed in a human adult are 1200 μm long, while those in minipigs are 350 μm long [49]. Nevertheless, their bone regeneration rate is comparable to that of humans (1.2–1.5 mm/day in minipigs vs. 1.0–1.5 mm/day in humans) [50], as is the rate of cortical bone mineralization, which is very similar between both species [51]. Even so, the present model also has a series of limitations, such as the fact that mesenchymal stem cells from minipigs have shown a significantly lower capacity to form differentiated and functional osteoblasts than those in humans [52]. Furthermore, there is a difference in the implantation site, since the mandibular or maxillary bone of humans is different in terms of structure and embryonic origin to the tibia of minipigs. However, it is considered that this animal model is suitable for this type of study, in which a fibroblast growth factor was immobilized on the surface of implants previously treated with carboxyethylphosphonic acid. It has been considered an important factor in research to evaluate the variations in biological reactions to growth factors in this species, which offers sufficient bone tissue for the placement of implants of a suitable size for use in humans. The choice of this 4-week healing period in minipigs was based on the study of Fuerst et al. in 2003, since, according to their results, a healing period of one month is sufficient to observe bone formation around dental implants treated with PDGF; furthermore, there was a significant increase in BIC and, therefore, in the mineralization of the interface of the bone on the implant surface [53]. However, the fact that no statistically significant differences were observed in the BIC parameter, in the densities or in the new bone formation in this study may be due to the insufficient healing period for the implants.

## 5. Conclusions

The application of carboxyethylphosphonic acid on the surface of implants can be considered a promising alternative as a biomimetic coating for the immobilization of FGF-2. In the pig model, the biofunctionalization with FGF-2 of the implant surface accelerated the mineralization of the bone–implant interface to a cortical level, thus reducing the period of osseointegration.

## Figures and Tables

**Table 1:** Representation of the results obtained for the BIC variable of the test and control groups.

Implant (Test Group)	Bone-Implant Contact (BIC)	Implant (Control Group)	Bone-Implant Contact (BIC)
C2TIMSP1	23.69%	C1TIMSE1	21.91%
C2TIDSP2	28.15%	C1TICSE1	27.02%
C4TIMSP3	42.60%	C1TIDSE3	23.55%
C4TICSP4	58.38%	C3TIMSE4	28.82%
C4TIDSP5	29.21%	C3TIDSE5	37.53%
C6TIMSP6	61.36%	C5TIMSE6	46.47%
C6TICSP7	65.34%	C5TICSE7	40.60%
C6TIDSP8	62.39%	C5TIDSE8	46.11%

**Table 2:** Representation of the results obtained for the BICc variable of the test and control groups.

Implant (Test Group)	Corrected Bone-Implant Contact (BIC_C_)	Implant (Control Group)	Corrected Bone-Implant Contact (BIC_C_)
C2TIMSP1	39.56%	C1TIMSE1	24.81%
C2TIDSP2	42.74%	C1TICSE2	40.94%
C4TIMSP3	57.85%	C1TIDSE3	37.46%
C4TICSP4	73.91%	C3TIMSE4	34.42%
C4TIDSP5	38.87%	C3TIDSE5	45.31%
C6TIMSP6	76.38%	C5TIMSE6	51.39%
C6TICSP7	71.15%	C5TICSE7	54.97%
C6TIDSP8	83.36%	C5TIDSE8	55.37%

**Table 3:** Representation of the results obtained for the BV/TV variable of the test and control groups.

Implant (Test Group)	New Bone Formation (BV/TV)	Implant (Control Group)	New Bone Formation (BV/TV)
C2TIMSP1	27.37%	C1TIMSE1	26.79%
C2TIDSP2	28.58%	C1TICSE1	20.71%
C4TIMSP3	33.03%	C1TIDSE3	24.48%
C4TICSP4	22.16%	C3TIMSE4	16.38%
C4TIDSP5	27.07%	C3TIDSE5	40.22%
C6TIMSP6	22.28%	C5TIMSE6	33.38%
C6TICSP7	31.55%	C5TICSE7	31.03%
C6TIDSP8	26.18%	C5TIDSE8	20.08%

**Table 4:** Results obtained for the variable BAI/TA of the test and control groups.

Implant (Test Group)	Interthread Bone (BAI/TA)	Implant (Control Group)	Interthread Bone (BAI/TA)
C2TIMSP1	19.14%	C1TIMSE1	39.15%
C2TIDSP2	26.44%	C1TICSE1	27.72%
C4TIMSP3	38.39%	C1TIDSE3	24.14%
C4TICSP4	35.88%	C3TIMSE4	22.52%
C4TIDSP5	37.16%	C3TIDSE5	36.22%
C6TIMSP6	37.35%	C5TIMSE6	45.28%
C6TICSP7	29.93%	C5TICSE7	35.59%
C6TIDSP8	33.87%	C5TIDSE8	32.64%
